# eHealth Delivery of Educational Content Using Selected Visual Methods to Improve Health Literacy on Lifestyle-Related Diseases: Literature Review

**DOI:** 10.2196/18316

**Published:** 2020-12-09

**Authors:** Azusa Aida, Thomas Svensson, Akiko Kishi Svensson, Ung-Il Chung, Toshimasa Yamauchi

**Affiliations:** 1 Precision Health Department of Bioengineering Graduate School of Engineering, The University of Tokyo Tokyo Japan; 2 Department of Diabetes and Metabolic Diseases Graduate School of Medicine The University of Tokyo Tokyo Japan; 3 Department of Clinical Sciences Lund University Skåne University Hospital Malmö Sweden; 4 School of Health Innovation Kanagawa University of Human Services Kawasaki-shi Japan; 5 Clinical Biotechnology Center for Disease Biology and Integrative Medicine Graduate School of Medicine, The University of Tokyo Tokyo Japan

**Keywords:** application, educational, eHealth, health literacy, lifestyle-related disease, mHealth, review

## Abstract

**Background:**

Lifestyle-related diseases, such as stroke, heart disease, and diabetes, are examples of noncommunicable diseases. Noncommunicable diseases are now the leading cause of death in the world, and their major causes are lifestyle related. The number of eHealth interventions is increasing, which is expected to improve individuals’ health literacy on lifestyle-related diseases.

**Objective:**

This literature review aims to identify existing literature published in the past decade on eHealth interventions aimed at improving health literacy on lifestyle-related diseases among the general population using selected visual methods, such as educational videos, films, and movies.

**Methods:**

A systematic literature search of the PubMed database was conducted in April 2019 for papers written in English and published from April 2, 2009, through April 2, 2019. A total of 538 papers were identified and screened in accordance with the PRISMA (Preferred Reporting Items for Systematic Reviews and Meta-Analyses) flow diagram. Finally, 23 papers were included in this review.

**Results:**

The 23 papers were characterized according to study characteristics (author and year of publication, study design and region where the study was conducted, study objective, service platform, target disease and participant age, research period, outcomes, and research method); the playback time of the educational videos, films, and movies; and the evaluation of the study’s impacts on health literacy. A total of 7 studies compared results using statistical methods. Of these, 5 studies reported significant positive effects of the intervention on health literacy and health-related measures (eg, physical activity, body weight). Although most of the studies included educational content aimed at improving health literacy, only 7 studies measured health literacy. In addition, only 5 studies assessed literacy using health literacy measurement tools.

**Conclusions:**

This review found that the provision of educational content was satisfactory in most eHealth studies using selected visual methods, such as videos, films, and movies. These findings suggest that eHealth interventions influence people’s health behaviors and that the need for this intervention is expected to increase. Despite the need to develop eHealth interventions, standardized measurement tools to evaluate health literacy are lacking. Further research is required to clarify acceptable health literacy measurements.

## Introduction

### Lifestyle-Related Diseases

Lifestyle-related diseases, such as stroke, heart disease, cancer, diabetes, and chronic respiratory disease, are leading examples of noncommunicable diseases and are now the leading causes of death in the world [1]. Of the top 10 causes of death worldwide in 2016, 6 are considered noncommunicable diseases and accounted for 71% of all deaths [2]. Lifestyle behaviors (eg, smoking, harmful consumption of alcohol, overeating, lack of exercise) or conditions (eg, overweight or obesity, hypertension, abnormal lipid metabolism, and hyperglycemia) are common risk factors for lifestyle-related diseases [3,4]. Lifestyle improvement (eg, high-quality diet, increased exercise) plays an important role in the prevention of lifestyle-related diseases [4,5], and improvement for some conditions can be achieved using eHealth-based interventions [3]. Furthermore, the association between lifestyle behavior and health literacy has been widely recognized [4,6].

### Health Literacy

Health literacy is defined as “people’s knowledge, motivation and competences to access, understand, appraise, and apply health information in order to make judgments and take decisions in everyday life concerning healthcare, disease prevention and health promotion to maintain or improve quality of life during the life course” [7]. Low health literacy is associated with poor health outcomes and delayed diagnosis and treatment [8]. For example, people with limited health literacy may experience a distrust of providers, pessimism about treatment, and poor satisfaction with the quality of care, probably due to communication difficulties (understanding verbal directions, signs, and placards as well as complexity of instructions) [8]. Occasionally, these patients find it difficult to navigate their way in health care facilities and are therefore unable to receive primary prevention [9]. Conversely, an improvement in health literacy is associated with better health outcomes, such as changes in risk for chronic disease, a reduction in reported disease severity, and decreases in the number of emergency department visits and hospitalizations [10]. Improving health literacy could even expand and extend people’s lives in the social, cultural, and work dimensions [7,11]. High rates of low health literacy in populations have prompted governments and national agencies in affected countries to develop national strategies and targets aimed at improving the health literacy of the general population [10].

### Information and Communication Technologies, eHealth, and Mobile Health

The popularity of mobile technologies remains high and the number of users of mobile technologies is increasing [12]. The growing usability of information and communication technologies (ICTs), including mobile apps and web-based applications, can increase the accessibility of health support systems [13-15]. eHealth includes medical information services, including public health services, that are distributed via the internet and related technologies [16]. Mobile health (mHealth) is an expanding area within eHealth and involves the use of mobile computing in the fields of medicine and public health [12]. The use of mHealth services, including smartphone-based services, may benefit health care providers by exerting positive effects on patient education, diagnosis, and management as components of the health delivery processes [12,15]. Smartphones provide a range of functions, including telephone calls, text messages (SMS), photos, video, and web access [12,17]. 

Educational content to improve individuals’ health literacy on lifestyle-related diseases can be offered in many ways, such as group learning, questionnaires, internet-based information searches, and downloadable apps [18-22]. Additionally, smartphone apps provide information through visual methods, such as graphics, videos, and pictures, which facilitate user understanding [14,21-23]. Many children and adults play video games, which include messages and entertaining formats and may lead users to change their health behaviors [18,24]. Accordingly, we wanted to investigate whether videos, games, and pictures are effective in increasing health awareness.

### Objectives

The aims of this review are to identify literature published in the past decade on eHealth interventions that aimed to improve health literacy on lifestyle-related diseases among the general population using selected visual methods, such as videos, films, and movies. Specifically, our review seeks to categorize study characteristics and the evaluation of the studies’ impacts on health literacy. Four main themes are discussed in this review: target age, measurement of health literacy, dietary health behavior, and the future directions of eHealth interventions.

## Methods

This literature review was performed using a systematic search and was conducted to emphasize the integration of studies across broader topics [3], with reference to PRISMA (Preferred Reporting Items for Systematic Reviews and Meta-Analyses) [25,26]. The search strategy was developed on April 2, 2019, by AA and TS, who also conducted the search.

**Figure 1:**
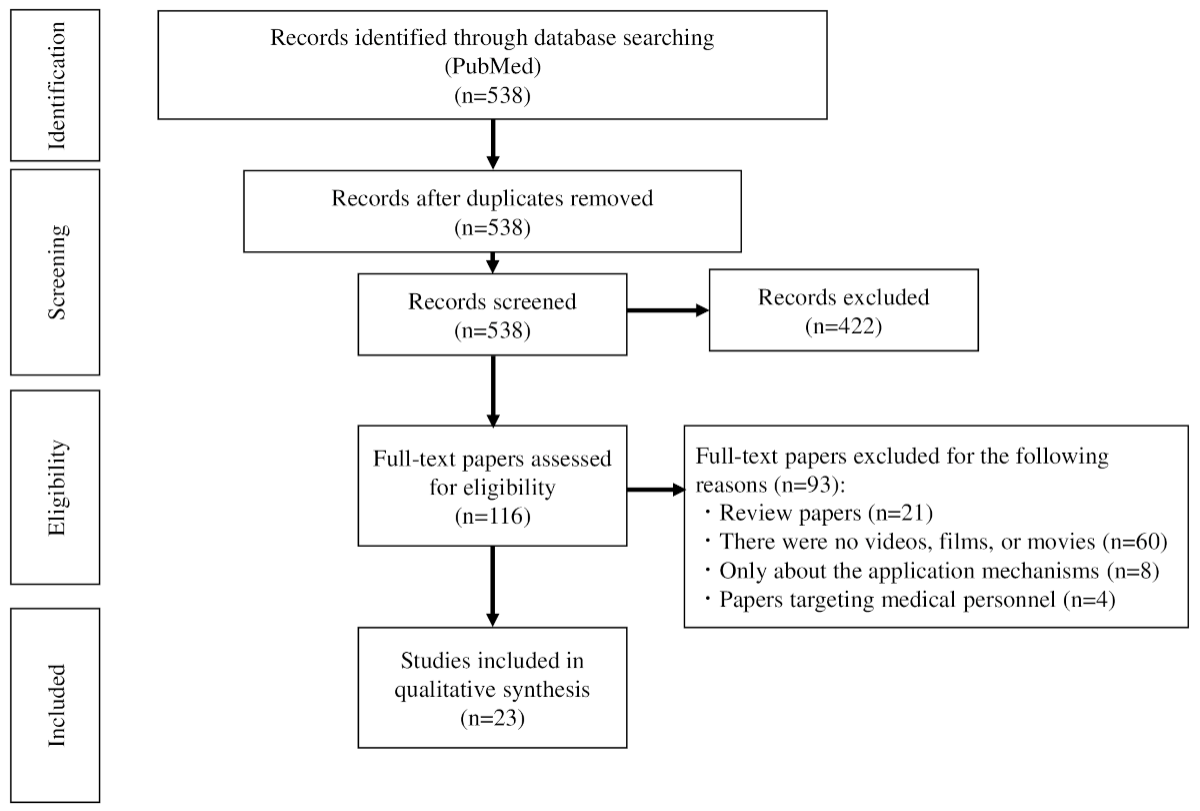
PRISMA flow diagram of the paper selection process. PRISMA: Preferred Reporting Items for Systematic Reviews and Meta-Analyses.

Details of the search strategy, study selection, and extraction of information can be found in Multimedia Appendix 1. In brief, PubMed was searched in the title and abstract fields using search terms that covered 5 main domains: “digital health,” “mHealth,” “education,” “health literacy,” and “visual methods.” The search identified 538 papers, with no duplicates (Figure 1). All papers were screened using a 2-stage process. In the first stage, 373 papers that did not meet the inclusion criteria were excluded. In the second stage, an additional 49 papers were excluded. The full texts of the 116 remaining papers were further surveyed, resulting in the exclusion of 93 papers, thereby leaving a total of 23 papers to review.

A data-charting form was created to categorize the included studies, the study characteristics (author and year of publication, study design and region where the study was conducted, study objective, target disease and participant age, research period, outcomes, and research method); the playback time of the educational videos, films, and movies; and the evaluation of the study’s impacts on health literacy (measurement to assess health literacy).

## Results

### Characteristics of the Included Studies

The included studies are characterized in Multimedia Appendix 2. Of the 23 papers we explored, 13 [18,27-38] were conducted in 2 stages, namely app development followed by intervention research. Two studies conducted randomized controlled trials (RCTs) [27,39], of which 1 [27] described the study protocol only. In addition, 8 studies were pilot studies [28-32,40-42], 6 studies were feasibility studies [33-38], 1 was an intervention study [43], and 1 was a cohort study using qualitative and quantitative methods [44]. As shown in Multimedia Appendix 3, primary outcomes were measured and the results were reported in 1 RCT [39], 1 intervention study [43], 5 pilot studies [30,31,40-42], and 2 feasibility studies [34,36]. A total of 7 studies [30,34,39-43] investigated the results statistically, of which 5 studies [31,34,39,40,43] reported significant positive effects on health literacy [34,39,43], and health-related measures (eg, physical activity, body weight) [30,41].

In addition, 3 noninterventional studies [18,45,46] were included because their main topics were video games, which was consistent with our search term of apps that included “games.”

### Study Objectives and Target Diseases

The objectives of the included studies were categorized into 3 groups: (1) health promotion, (2) disease prevention, and (3) disease management related to the target diseases.

One study [40] was conducted for health promotion and was an innovative mHealth cardiovascular health promotion program, 9 studies [18,27,30,33-35,45-47] aimed to prevent disease by reducing the risks for the diseases, 4 studies [18,34,45,46] promoted smoking cessation for smokers, 2 studies promoted weight loss to address obesity [30,33], 2 studies promoted diet and exercise to lower blood glucose levels in order to manage prediabetes [27,35], and 1 study [47] promoted physical activity to prevent lifestyle-related diseases.

A total of 15 studies [27-29,31,32,35-39,41-44,48] aimed to manage a disease, including diabetes (n=6) [27,31,35,38,42,43] (1 study targeted type 1 diabetes [42] and the remainder targeted type 2 diabetes [27,31,35,43,47]), heart failure (n=3) [41,44,48], cardiovascular disease (n=2) [28,32], stroke (n=2) [36,37], gout (n=1) [29], chronic obstructive pulmonary disease (n=1) [38], and osteoarthritis of the knee (n=1) [39].

### Outcomes

Outcomes were investigated in 18 studies [18,27,29-36,38-44,48] and categorized into 2 types: (1) changes in measured values and (2) changes in dietary health behaviors.

Studies used a number of measured values: body weight [27,29,30,33,41,44,48], waist circumference [27,30], blood pressure [27,44], oxygen usage [38,44], hemoglobin A_1c_ [27,38,42], serum uric acid [29], and knowledge about health conditions (eg, diabetes knowledge, self-efficacy score, cardiovascular health knowledge, smoking knowledge) [31,34,39,40,42,43]. Studies also investigated behavioral changes, smoking attitude [18,34], nutrition [33,35,38,40], physical activity [33,35,38,41], stress [33], medication [32,38], specific health behavior [27], and usability and acceptability of the intervention [30,36].

To assess changes in measured values, individuals’ health literacy was tested in 7 studies [31,34,39,40,42,43,48], and measurement tools were used in 5 studies only [31,34,40,42,43]. A 27-item questionnaire based on the Norwegian National Health Informatics' diabetes quiz to test theoretical knowledge was conducted for individuals aged 13 to 19 years with type 1 diabetes. The eHealth Literacy Scale (eHEALS) [40] and smoking knowledge score [34] were used for participants 18 years or older. The Rapid Estimate in Adult Literacy in Medicine (REALM) [31], the Diabetes Self-Efficacy Scale [31], and 2 types of study knowledge tests [39,48] were used for participants older than 40 years.

### Platform of Development of eHealth Service

We categorized the included studies into 3 types of platforms: (1) applications (web-based applications or mobile apps), (2) websites, and (3) others.

Various apps were used in 22 studies [18,27-37,39-48]. Apps allow users to use interactive content [30,32,39,43], telephone interviews [44], face-to-face video conferencing [33,40,44], and social network service messages [42]. User satisfaction was evaluated for the intervention itself and for opinions on future development in 16 studies [18,30-32,34-37,39,41,42,44-48]. The 14 apps [18,28-32,34,37,39,41,42,44,45,48] receiving the highest satisfaction and appreciation ratings included those providing educational content (about diseases [30-32,34,39,41,48], through a diary program [29,42], and in game content [18,45,46]) using pictures, graphics [27,29,30,34,37,42,44], videos [18,27-31,33-37,39,41-45,47], icons, drawings, animations [47], and an avatar [48]. Only one app providing educational video [30] was considered unacceptable because the process of downloading and viewing the video was too difficult or took too much time.

By connecting with a wearable device [28], sensing devices (eg, blood pressure monitor, weight scales, pulse oximeter) [44], and Bluetooth [42], health information (eg, physical activity, blood glucose values) could be provided remotely.

Websites were based on a textual design to provide educational messages but, unlike apps, were unable to include a gaming component to provide some type of feedback-based reward [45].

Another platform [38] developed internet-enabled home programs that provided educational videos, individual consultations, and a health diary remotely using the patient’s television at home and a connection to a computer.

## Discussion

In this review, we aimed to identify and characterize the features of existing literature describing eHealth interventions that aimed to improve health literacy on lifestyle-related diseases among the general population using selected visual methods, such as videos, films, and movies. Through this research, we identified 4 themes, which we discuss here with their strengths and limitations.

### Target Age

We found a difference in target age compared with previous studies focused on health literacy. Kim and Xie [14] found that health literacy was limited in individuals older than 65 years. In this review, 19 studies [27-36,38-42,44,45,47,48] included participants younger than 65 years. Although it appears that people with low health literacy are older [49,50], young adults also lack health literacy, including eHealth literacy [51,52].

In our review, we identified various ideas to facilitate the use of educational content to improve participants’ health literacy and found that the kind of educational content provided was required to change as the age range of the target population widened. The use of icons and avatars facilitated usage for both younger and older individuals with low health literacy [41,48]. The younger the target age, the greater the acceptability of games [46].

### Measurement of Health Literacy

Measurement tools for health literacy have yet to be established. Indeed, in this review, among the 22 included studies that developed apps for education [18,27-37,39-48], only 5 studies [31,34,40,42,43] used instruments to measure health literacy.

According to a review [14] that identified the relevant literature and examined the instruments used to measure individual health literacy levels, most studies used the eHEALS [53] and the Short Test of Functional Health Literacy (S-TOFHLA) [54]. The S-TOFHLA is a shortened version of the Test of Functional Health Literacy [55], which correlates with the REALM [56]. In another systematic review of health literacy using web-based health information environments [26], the Newest Vital Sign was used most often [57], followed by the REALM [58]. There is a lack of standard measurement tools to evaluate health literacy [17,22,59-62]. Additional research is required to identify measurement methods suitable for evaluating levels of health literacy.

### Dietary Health Behavior Change

Improvements to lifestyle play an important role in lifestyle-related diseases [4,5]. Although 12 included studies [18,27,30,32-36,38,40,41,44] had changes in dietary health behavior as an outcome, none described the mechanism of the relationship between the eHealth intervention and the dietary health behavior. One study [32] classified an educational interventional app in terms of behavior change techniques (BCTs) used in behavior change interventions. Future research to investigate the underlying mechanism of BCTs will be useful in clarifying which interventions are likely to be effective.

### Future Directions of eHealth Interventions

Based on user evaluations of the interventions and opinions on their future development, eHealth interventions using visual methods and interactive approaches improve user motivation to improve lifestyle and health literacy. However, 1 study [30] reported that the use of educational videos was not acceptable due to the difficulty older participants experienced in using the mobile app. Future eHealth-based interventions will be required to improve users’ computer literacy and identify the mediating effects of age and sex.

### Strengths and Limitations

To our knowledge, this study is the first to review the educational content of eHealth aimed at improving individuals’ health literacy on lifestyle-related diseases. Our findings may provide a new perspective on the development of apps that use eHealth to address lifestyle-related diseases and improve people’s health literacy.

Several limitations of our study warrant mention. One limitation is the search process: we searched only a single database and included only studies written in English and published in the decade up to April 2019. As discussed in other review papers about eHealth-based interventions [3,14], selecting from additional databases that include unpublished studies is useful for a broader review and should be a consideration for any future systematic review on the topic. Nevertheless, the search was conducted in a systematic manner using the PRISMA flow diagram [25,26]. Additionally, we excluded review papers; papers about AIDS, cancer, psychiatric conditions, odontology, and pediatrics; papers whose target populations were medical personnel and pregnant women; and papers that did not include educational videos, films, or movies. This strategy may have been too strict and some relevant papers may have been missed. Future reviews should be based on wider search criteria.

### Conclusions

Our review provides an overview of the relationships between eHealth-based interventions with selected visual methods, such as videos, films, and movies, and improved health outcomes (ie, changes in measured values and dietary health behavior). Despite the necessity of self-management systems using ICT to control lifestyle-related diseases, relatively few studies have explored educational videos, films, and movies aimed at improving health outcomes.

We also found that the concept of literacy and the tools used to measure the outcome of health literacy–related interventions have not been unified. To more accurately evaluate levels of health literacy and the effects of interventions, future studies need to clarify the concept of health literacy and develop health literacy screening tools.

## Appendix

Multimedia Appendix 1 Supplementary methods.

Multimedia Appendix 2 Characteristics of the included studies.

Multimedia Appendix 3 Summary of the study design, intervention, and the results of the included randomized controlled trial (RCT), intervention study, pilot studies, and feasibility studies.

## Abbreviations

BCT: behavior change technique
eHEALS: eHealth Literacy Scale
ICT: information and communication technology
mHealth: mobile health
RCT: randomized controlled trial
REALM: Rapid Estimate in Adult Literacy in Medicine
S-TOFHLA: Short Test of Functional Health Literacy
