# Supplementary material for: eHealth Delivery of Educational Content Using Selected Visual Methods to Improve Health Literacy on Lifestyle-Related Diseases: Literature Review
Source: JMIR Mhealth Uhealth. 2020 Dec 9;8(12):e18316. doi: 10.2196/18316 (PMC7758165; doi:10.2196/18316)
Supplement: Multimedia Appendix 1 [file mhealth_v8i12e18316_app1.docx]

**Multimedia Appendix 1**

Contents:

Supplementary Methods (page 1-2)

References (page 3)

**SUPPLEMENTARY METHODS**

**Data sources and search strategy**

PubMed was used for the search queries. Inclusion was limited to articles published in the ten years (between 2009 and April 2nd, 2019) preceding the search. This date range was chosen due to the relatively recent emergence of eHealth in the 21st century [1] and the increasing use of mobile devices and online services [1, 2].
PubMed was searched in the title and abstract fields using the following search terms: (((mobile AND (application OR "app" OR apps)) OR "mHealth") OR ("digital health")) AND ((educational AND (film OR "video" OR movie OR contents OR information)) OR (health literacy)). Scholarly publications, including articles in peer-reviewed journals, were searched in PubMed, and 538 articles were identified.

**Study Selection and Extraction of information**

Articles were screened using a two-stage process. In the first stage, the titles of 538 articles were screened based on the following criteria: Inclusion criteria were (1) original papers, including peer-reviewed journal articles; (2) studies focused on health literacy; (3) study designs were clinical trials, with a broad range of methodology papers including qualitative and quantitative; (4) any eHealth intervention using visual methods such as videos, films, and movies to influence user’s health literacy, health behavior, health outcome; (5) studies targeting people with unhealthy lifestyle behaviors or conditions (e.g., smoking, obesity, and hyperglycemia) regardless of age, gender, or ethnicity; and (6) full text in English. Concerning improvement in health literacy and health behavior change, papers describing a change in health literacy, health status (e.g., body weight, waist circumference), and biochemical results (e.g., uric acid, blood glucose level) were also included.

Exclusion criteria were (1) review papers or case reports; (2) targeted populations were medical personnel (e.g., doctors, nurses, pharmaceutical chemists), medical students, or expectant mothers; (3) populations with specific diseases and disorders such as Acquired Immunodeficiency Syndrome (AIDS), cancer, or psychiatric conditions; (4) studies within the fields of odontology, and pediatrics; and (5) papers limited to application mechanisms, as their main focus was from an engineering point-of-view. A total of 373 articles that did not meet the inclusion criteria were excluded. In the second stage, the abstracts of the 165 remaining articles were screened using the same search criteria as in the first stage, and a total of 49 articles were excluded. In total, 422 articles were excluded in this two-stage process screening.

The full texts of the 116 remaining articles were further surveyed for whether each study met our inclusion criteria. Finally, a total of 93 articles were excluded because they were review papers (n=21), did not use the visual methods (i.e., videos, films or movies) within the scope of this review (n=60), were limited to a description of the application mechanism (n=8), or targeted medical personnel (n=4). Finally, a total of 23 articles were included and examined in detail (Figure 1).

**References**

1. Stellefson M, Hanik B, Chaney B, Chaney D, Tennant B, Chavarria EA. eHealth literacy among college students: a systematic review with implications for eHealth education. J Med Internet Res. 2011 Dec;13(4):e102. PMID: 22155629. doi: 10.2196/jmir.1703.

2. Borzekowski DL, Leith J, Medoff DR, Potts W, Dixon LB, Balis T, et al. Use of the internet and other media for health information among clinic outpatients with serious mental illness. Psychiatr Serv. 2009 Sep;60(9):1265-8. PMID: 19723745. doi: 10.1176/ps.2009.60.9.1265.
