# Supplementary material for: eHealth Delivery of Educational Content Using Selected Visual Methods to Improve Health Literacy on Lifestyle-Related Diseases: Literature Review
Source: JMIR Mhealth Uhealth. 2020 Dec 9;8(12):e18316. doi: 10.2196/18316 (PMC7758165; doi:10.2196/18316)
Supplement: Multimedia Appendix 2 [file mhealth_v8i12e18316_app2.docx]

Multimedia Appendix 2. Characteristics of the included studies

| **Author,**  **year of**  **publication** | **Study design,**  **region where the study was conducted** | **Service platform** | **Study objective** | **Target disease or condition,**  **Participants’ age** | **Research period** | **Outcomes** | **Research method** | **Measurement of health literacy** | **Playback time of educational content** |
| --- | --- | --- | --- | --- | --- | --- | --- | --- | --- |
| Ceasar, J. N. et al, 2019 [28] | Pilot study, USA | Mobile application (app) | Disease management | Cardiovascular disease (CVD), n=16, age 19-85 years | More than 3 months | N/A | Questionnaire survey | N/A | N/A |
| Timmers, T. et al, 2018 [39] | RCT, Netherlands | Mobile app | Disease management | Knee osteoarthritis, n=213, age older than 40 years | 4 months | Changes in measured values: the level of perceived and actual knowledge about knee complaints, relevant treatment options | Questionnaire survey | Numeric Rating Scale, Linkert scale, patients' knowledge test | N/A |
| Nguyen, A. D. et al, 2018 [29] | Descriptive qualitative study, Australia | Mobile app | Disease management | Gout, Study 1: n=11, age 32-85 years, Study 2: n=5, age 50-69 years | More than 4 months | Changes in measured values: uric acid, weight | Questionnaire survey | N/A | 2 minutes (min) |
| Materia, F. T. et al, 2018 [33] | Feasibility study, USA | Mobile app | Disease prevention | Overweight and obesity, n=40, age 18-35 years | 3 months | Changes in measured values: weight, Changes in dietary health behaviors: nutrition, physical activity, stress | Group interview | N/A | N/A |
| Alanzi, T et al, 2018 [43] | Intervention study, Saudi Arabia | Mobile app | Disease management | Type 2 diabetes, n=82, age not mentioned | 2 months | Changes in measured values: diabetes knowledge, self-efficacy score | Questionnaire survey | Diabetic knowledge test (DKTest), diabetic　management　self-efficacy test (DMSES) | N/A |
| Sarfati, D. et al, 2018 [27] | RCT (protocol), New Zealand | Web-based app, mobile app | Disease prevention, disease management | Type 2 diabetes and prediabetes, n=430, age 18-75 years | 1 year | Changes in measured values: HbA1c, weight, waist circumference, blood pressure, Changes in dietary health behaviors: self-management, score in diabetic-specific behaviors | Questionnaire survey, task data | N/A | N/A |
| Brewer, L. C. et al, 2018 [40] | Pilot study, USA | Mobile app | Health promotion | CVD, n=50, aged 18 years or older | More than 6 months | Changes in measured values: self-efficacy, cardiovascular (CV) health knowledge, Changes in dietary health behaviors: nutrition | Questionnaire survey | Self-efficacy, CV health knowledge, eHealth Literacy Scale (eHEALS) | N/A |
| Lloyd, T. et al, 2019 [41] | Pilot study, USA | Web-based, mobile app | Disease management | Heart failure (NYHA class 2-3), n=12, age 52-74.5 years | 3 months | Changes in measured values: weight, Changes in dietary health behaviors: physical activity | Questionnaire survey, task data | N/A | 5 min |
| Bindoff, I. et al, 2016 [18] | The design of app before RCT, Australia | Mobile app | Disease prevention | Smokers, n=7 (iOS), n=11 (Android), age not mentioned | 1 month | Changes in dietary health behaviors: smoking attitude | Questionnaire survey | N/A | 1-5 min |
| Fontil, V. et al, 2016 [35] | Adaptation, Feasibility study, USA | Web-based, mobile app | Disease prevention, disease management | Diabetes, prediabetes, n=64, age 18-75 years | More than 4 months | Changes in dietary health behaviors: nutrition, physical activity | Questionnaire survey | N/A | N/A |
| Finkelstein, J. et al, 2016 [34] | Feasibility study, USA | Mobile app | Disease prevention | Smokers, n=55, aged 18 years or older | N/A | Changes in measured values: smoking knowledge score (KS), Changes in dietary health behaviors: smoking attitude | Questionnaire survey | KS | N/A |
| Watson, A. M. et al, 2016 [45] | Content analysis, USA | Websites, web-based, mobile app | Disease prevention | Smokers, n=129 (games included for analysis), age not mentioned | N/A | N/A | Online search | N/A | 15 min |
| Choo, S. et al, 2016 [30] | Pilot study, Korea | Mobile app | Disease prevention | Obesity, n=30, age 20-70 years | More than  3 months | Changes in measured values: weight, abdominal circumference, Changes in dietary health behaviors: usability, acceptability, early effect on patient-doctor relationship | Questionnaire survey | N/A | 1.5 min |
| SureshkumarK. et al, 2016 [36] | Feasibility, Acceptability study, India | Websites, web-based, mobile app | Disease management | Stroke, n=60, age older than 18 years | 6 weeks | Changes in dietary health behaviors: operational difficulties, feasibility, acceptability | Questionnaire survey | N/A | N/A |
| Athilingam, P. et al, 2016 [48] | Beta testing, USA | Mobile app | Disease management | Heart failure, n=10, age 43-81 years | N/A | Changes in measured values: weight | Questionnaire survey | Knowledge test | N/A |
| Wood, F. G. et al, 2015 [31] | Pilot study, USA | Mobile app | Disease management | Type 2 diabetes, n=7, 43-64 years | 1 month | Changes in measured values: Health literacy, self-care activities, self-efficacy, diabetes knowledge | Questionnaire survey | Rapid Estimate in Adult Literacy in Medicine (REALM) score, Diabetes Knowledge Test, Diabetes Self-Efficacy Scale | N/A |
| SureshkumarK. et al, 2015 [37] | Intervention content, Feasibility study, India | Websites, web-based, mobile app | Disease management | Stroke, n=50 (patients and their caregivers), age not mentioned | N/A | N/A | Questionnaire survey | N/A | 3-5 min |
| Boyd, A. D. et al, 2015 [32] | Patient-centered design methodology, USA | Mobile app | Disease management | Patients underwent PCI and received a drug-eluting stent, n=6, age older than 50 years | 3 weeks | Changes in dietary health behaviors: medication adherence | Questionnaire survey | N/A | 17.6 min |
| Triantafyllidis,A. et al, 2015 [44] | Cohort study, Qualitative, Quantitative methods, UK | Mobile app | Disease management | Heart failure, n=26, age 18 years and above | 1 year | Changes in measured values: blood pressure, weight, pulse oximeter, Changes in dietary health behaviors: engagement with the system | Questionnaire survey, data from available sensing devices | N/A | N/A |
| Bender, M. S. et al, 2015 [47] | Qualitative study, USA | Mobile app | Disease prevention | Lifestyle-related diseases, n=16, age promoters: 41±17.0 years, Health Care Professionals: age 42±13.3 years | More than 3 months | N/A | Questionnaire survey | N/A | 2 min |
| BinDhim, N. F. et al, 2015 [46] | Research, Australia | Mobile app | Disease prevention | Smokers, n=107, age not mentioned | 10 months | N/A | N/A | N/A | N/A |
| Burkow, T. M. et al, 2013 [38] | Preliminary study, Norway | Websites | Disease management | Chronic obstructive pulmonary disease (COPD), diabetes, n=10, age COPD: 45-74 years, diabetes: 55-74 years | 14 weeks | Changes in measured values: oxygen usage, oxygen saturation, HbA1c, Changes in dietary health behaviors: medication, physical activity, nutrition | Questionnaire survey, task data | N/A | 10-40 min |
| Frøisland, D. H. et al, 2012 [42] | Pilot tested Mixed-methods study, Norway | Mobile app | Disease management | Type 1 diabetes, n=12, age 13-19 years | 3 months | Changes in measured values: HbA1c, diabetes knowledge | Questionnaire survey, data by Bluetooth technology | Theoretical knowledge as tested by a 27-item questionnaire based on the Norwegian National Health Informatics' diabetes quiz | N/A |

Abbreviations: app, application; CV, cardiovascular; CVD, Cardiovascular disease; DKTest, Diabetic knowledge test; DMSES, diabetic management self-efficacy test; eHEALS, eHealth Literacy Scale; KS, smoking knowledge score; min, minutes; NYHA, New York Heart Association; PCI, percutaneous coronary intervention; RCT, randomized controlled trial; REALM, Rapid Estimate in Adult Literacy in Medicine.
