# Supplementary material for: eHealth Delivery of Educational Content Using Selected Visual Methods to Improve Health Literacy on Lifestyle-Related Diseases: Literature Review
Source: JMIR Mhealth Uhealth. 2020 Dec 9;8(12):e18316. doi: 10.2196/18316 (PMC7758165; doi:10.2196/18316)
Supplement: Multimedia Appendix 3 [file mhealth_v8i12e18316_app3.docx]

Multimedia Appendix 3. Summary of the study design, intervention, and the results of the included randomized controlled trial (RCT), intervention study, pilot studies, and feasibility studies

| **Study** | **Study design** | **Disease, condition** | **Primary outcome** | **Period** | **Results** |
| --- | --- | --- | --- | --- | --- |
| Timmers, T. et al, 2018 [39] | RCT | Knee osteoarthritis | The level of perceived and actual knowledge　about knee complaints, relevant treatment options | 4 months | The level of actual knowledge: significantly higher in the application (app) group (p< 0.001), the level of perceived knowledge: significantly increased within the app group (p<0.001) |
| Alanzi, T et al, 2018 [43] | Intervention study | Type 2 diabetes | Diabetes knowledge, self-efficacy score | 2 months | Intervention group: knowledge, self-efficacy: significantly improved (p<0.001) |
| Brewer, L. C. et al, 2018 [40] | Pilot study | Cardiovascular disease (CVD) | Self-efficacy, cardiovascular (CV) health knowledge, CV health | More than  6 months | Participants had high eHealth Literacy (EHL) scores, no differences by sex (p=0.75) |
| Lloyd, T. et al, 2019 [41] | Pilot study | Heart failure | Weight, physical activity | 3 months | Patients reduced their weight at a rate of 0.17 lbs per day (95% CI: (-0.26, -0.08); p=0.002), slightly increased the activity time at a rate of 0.08 min per day ((95% CI: (0.004, 0.15); p=0.04) |
| Finkelstein, J. et al, 2016 [34] | Feasibility study | Smokers | Smoking knowledge score (KS), smoking attitude | N/A | Mean KS increased (p<0.0001), the proportion of patients who felt they “cannot quit smoking” reduced (p<0.03), the main factors affecting knowledge significantly gained, initial knowledge level (p<0.02), employment status (p<0.05), high app acceptance (p<0.01) |
|  |  |  |  |  |  |
| Choo, S. et al, 2016 [30] | Pilot study | Obesity | Usability, acceptability, early effect on patient-doctor relationship | More than 3 months | The median number of log-ins per day: 1.21, scales of the depth of the patient-doctor relationship significantly decreased from SD 4.8 to SD 4.5 (p=0.02) |
| Sureshkumar, K. et al, 2016 [36] | Intervention content, Feasibility study | Stroke | Operational difficulties, feasibility, acceptability | 6 weeks | Over 90% of the participants felt that the intervention was relevant, comprehensible, and useful. Over 96% of the stroke survivors and all the caregivers rated the intervention as excellent and very useful. |
| Wood, F. G. et al, 2015 [31] | Pilot study | Type 2 diabetes | Health literacy, self-care activities, self-efficacy, diabetes knowledge | 1 month | Rapid Estimate of Adult Literacy in Medicine (REALM) score: 45 to 65.5, Diabetes Knowledge Test: 15.5 to 16.5, Diabetes Self-Efficacy Scale: 6.7 to 8.1 |
| Frøisland, D. H. et al, 2012 [42] | Pilot tested Mixed-methods study | Type 1 diabetes | HbA1c, diabetes knowledge | 3 months | Not statistically significant, HbA1c (p=0.38), diabetes knowledge (p=0.82) |

Abbreviations: app, application; CI, Confidence Interval; CV, cardiovascular; CVD, Cardiovascular disease; EHL, eHealth Literacy; KS, Smoking knowledge score; RCT, randomized controlled trial; REALM, Rapid Estimate of Adult Literacy in Medicine; SD, standard deviation.
